# Are the Therapeutic Effects of Huangqi (*Astragalus membranaceus*) on Diabetic Nephropathy Correlated with Its Regulation of Macrophage iNOS Activity?

**DOI:** 10.1155/2017/3780572

**Published:** 2017-11-08

**Authors:** Hui Liao, Ling Hu, Xingnuo Cheng, Xiaocheng Wang, Jiarui Li, Linda Banbury, Rongshan Li

**Affiliations:** ^1^Department of Pharmacy, Shanxi Provincial People's Hospital, Shanxi Medical University, Taiyuan 030012, China; ^2^Department of Endocrinology, Shanxi Provincial People's Hospital, Shanxi Medical University, Taiyuan 030012, China; ^3^School of Pharmacy, China Pharmaceutical University, Nanjing 211198, China; ^4^Department of Medical Record & Statistics, Shanxi Provincial People's Hospital, Shanxi Medical University, Taiyuan 030012, China; ^5^State Key Laboratory of Genetic Engineering, School of Life Sciences, Fudan University, Shanghai 200438, China; ^6^School of Health and Human Sciences, Southern Cross University, Military Rd, Lismore, NSW 2480, Australia; ^7^Department of Nephrology, Shanxi Provincial People's Hospital, Shanxi Medical University, Shanxi Kidney Disease Institute, No. 29 Shuangtasi Street, Taiyuan, Shanxi 030012, China

## Abstract

**Objective:**

To investigate the correlation between the clinical effects of Huangqi (*Astragalus membranaceus*) on different stages of diabetic nephropathy (DN) and the pharmacological effect of Huangqi on the activity of inducible nitric oxide synthase (iNOS) in macrophages in different states.

**Methods:**

The PubMed, China National Knowledge Infrastructure, and Wanfang databases were searched. Clinical data was sourced from papers on treatment of different stages of DN with Huangqi, and pharmacological data was from papers on the effects of Huangqi on the iNOS activity of macrophages in a resting or an activated state.

**Results:**

Meta-analysis of Huangqi injections on stages III and III-IV DN and randomized controlled trials on other stages showed that Huangqi had therapeutic effects on different stages of DN and on macrophages in different states: inducing normal macrophages in a resting state to generate nitric oxide (NO), tumor necrosis factor-*α*, and so forth upon iNOS activation; inhibiting NO generation by normal lipopolysaccharide- (LPS-) activated macrophages; and enhancing NO generation by LPS-induced macrophages from patients with renal failure.

**Conclusions:**

Huangqi can regulate iNOS activity of macrophages in different states *in vitro*. These biphasic or antagonistic effects may explain why Huangqi can be used to treat different stages of DN.

## 1. Introduction

Diabetic nephropathy (DN) is one of the most serious chronic microvascular complications of diabetes mellitus (DM) and is also the main cause of renal failure in end-stage chronic kidney disease (CKD). Mogensen et al. divide DN into five stages, according to the course and pathophysiological process of the disease (Table 1) [1]. Proteinuria is a hallmark of diabetic kidney disease and is also an independent risk factor for the progression of renal failure [2]. A pharmacological study showed that Huangqi (*Astragalus membranaceus*), a traditional Chinese medicine (TCM), attenuates proteinuria in a streptozotocin- (STZ-) induced model of diabetes [3].

Recent research showed that there are significant differences between urinary mRNA of podocyte-associated molecules in relation with albuminuria stage [4], and the activation of macrophages causes podocyte damage in DN [5]. Macrophages in a resting state appear to have no obvious effect on renal injury, and macrophages in an activated state are very important to the disease progression. After inducible nitric oxide synthase (iNOS, nitric oxide synthase type II) is induced by lipopolysaccharide (LPS), high glucose, or the like, a large amount of nitric oxide (NO) is generated, a marker of macrophage activation [6].

There has been extensive research on Huangqi in the clinical treatment of DN, and Huangqi has been shown to exhibit particular therapeutic effects on different stages of DN [7–28]. Existing studies have shown that Huangqi has an inhibitory effect on the generation of NO by LPS-induced macrophages [29–42] and also that Huangqi itself can induce macrophages to generate NO [41–49]. This article attempts to analyze the correlation between these apparently antagonistic pharmacological effects of Huangqi on macrophages and the clinical effect of Huangqi in the treatment of different stages of DN, based on existing clinical data and the results of relevant pharmacological studies.

## 2. Materials and Methods

### 2.1. Literature Retrieval Strategy

The Chinese journal full-text database of China National Knowledge Infrastructure (CNKI), Wanfang database (Wanfang), and Medline were electronically searched, from the inception of the databases until August 2017. (*Astragalus membranaceus* OR Huangqi) AND (diabetic nephropathies OR renal failure) were selected as MeSH terms for clinical data. (*Astragalus membranaceus* OR Huangqi) AND macrophages AND nitric oxide synthase type II were selected as the MeSH terms for pharmacological studies.

### 2.2. Incorporation of Literature

Clinical data was incorporated from articles satisfying the following criteria: papers that demonstrate the therapeutic effects of Huangqi on different stages of DN, involving randomized controlled trial (RCT), semirandomized controlled trial (CCT), and meta-analyses of RCTs and CCTs. In such data, the DM should be diagnosed according to the diagnostic criteria of the WHO (1980, 1985, or 1999) or the American Diabetes Association (1997 or 2010), and the stages of DN should be diagnosed according to Mogensen's criteria for stage diagnosis [1].

Pharmacological studies were incorporated from articles satisfying the following criteria: *in vitro* pharmacological studies on the influence of Huangqi on the generation of NO, tumor necrosis factor-*α* (TNF-*α*), and so forth by macrophages upon iNOS expression. The macrophages include normal macrophages and immunocompromised macrophages, in either resting state or activated state.

## 3. Results

### 3.1. Preliminary Analysis of Clinical Studies on Treatment of DN with Huangqi

In this study, CNKI was initially used for a preliminary analysis of the clinical reports of treatment of different stages of DN with Huangqi. The retrieval results show that therapeutic effects of Huangqi on all stages of DN have been reported. 484 clinical research papers relate to treatment of different stages of DN with Huangqi, with the majority of these (243) referring to Huangqi injections.

### 3.2. Clinical Studies on Treatment of Different Stages of DN with Huangqi Injections

**Figure 1:**
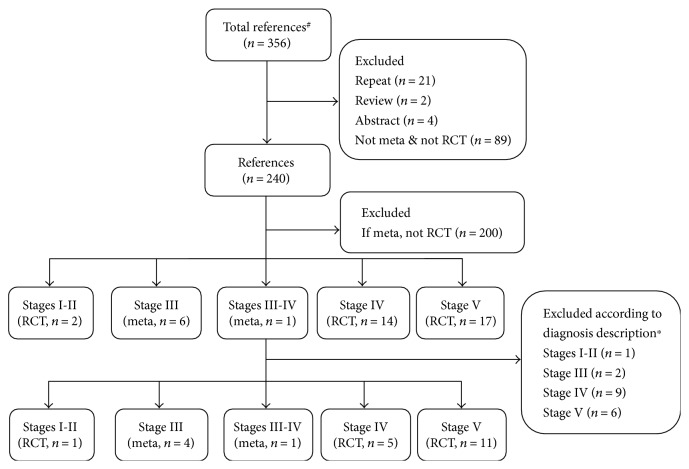
Flowchart on clinical trials. *Note*. ^#^Total references included 243 from the Chinese journal full-text database of China National Knowledge Infrastructure and 113 from Wanfang database. ^∗^The diabetes mellitus should be diagnosed according to the diagnostic criteria of the WHO (1980, 1985, or 1999) or the American Diabetes Association (1997 or 2010), and the stages of diabetic nephropathy should be diagnosed according to Mogensen's criteria for stage diagnosis [1].

Based on the preliminary retrieval results of Section 3.1, Medline, Wanfang, and CNKI were searched. The papers involving treatment of DN with Huangqi injections were classified, collected, and summarized according to different stages: meta-analyses of therapeutic effects on different stages were first selected, and then relevant RCT or CCT studies were selected, so as to clarify the therapeutic effects of Huangqi injections on the five stages of DN. As shown in the flowchart in Figure 1, ultimately 22 references were chosen from a total of 356 references, including 5 meta-analyses and 17 RCTs [7–28].

There was only one RCT report about Huangqi injections against stages I-II of DN, with a relatively small sample size (28 in total, 13 in control group and 15 in Huangqi injection treatment group). The main improvement indicators reported on included urine albumin excretion rate (UAER), transforming growth factor *β*1 (TGF-*β*1), amongst others [7].

There are four meta-analyses that demonstrate the therapeutic effects of Huangqi injections on stage III DN. The studies incorporated into these four meta-analyses on stage III were conducted from 1998 to 2015, including 49 RCTs (29 RCTs were cited in more than one meta-analysis). The total cases were 3368 in which 1644 cases were in the control group and 1724 in the treatment group. UAER improved in these four meta-analyses. Both 24-hour urinary protein and serum creatinine (Scr) were reported in three analyses, and blood urea nitrogen (BUN) in two [8–11].

One meta-analysis related to stages III-IV was reported in 2011, including 21 RCTs and 4 CCTs [12]. A report about Huangqi injections at stage IV included five RCTs. The total cases were 455 (199 in the control group and 256 in the treatment group [13–17]). The improvement indicators included 24-hour urinary protein, Scr, BUN, and creatinine clearance rate (CCr).

An RCT report about stage V included 32 cases in the treatment group and 30 in the control group [18]. There are another 10 RCT papers related to renal failure research using Huangqi injections. We extracted 210 cases of renal failure due to DN (120 cases in the treatment group and 90 cases in the control group) from a total of 1372 cases [19–28]. All these were included in Table 2 stage V, and Scr, BUN, and CCr were improved [18–28].

### 3.3. Pharmacological Studies on Regulation of iNOS Activity of Macrophages in Different States by Huangqi

There are 21 articles about the influence of Huangqi on the generation of NO, TNF-*α*, and so forth by normal macrophages upon iNOS expression *in vitro* [29–49], including 9 in Chinese [29–32, 41–45], and 12 in English [33–40, 46–49], as shown in Table 3. The macrophages used in the studies were derived from either RAW264.7 macrophage cell line, peritoneal macrophages, or human mononuclear macrophage line U937.

Twelve articles reported that Huangqi could inhibit the generation of NO by LPS-activated macrophages [29–40]; eight studies were performed on Huangqi extracts, including crude extracts, active fractions, and compounds [31, 34–40], two studies on polysaccharides [29, 30], and another two on saponins and total flavonoids separately [32, 33]. Seven articles separately reported that Huangqi could induce normal macrophages in a resting state to generate NO, focusing on polysaccharides and saponins, with polysaccharides (6 studies) being the most studied [43–49]. Two articles reported that Huangqi could both induce the generation of NO by macrophages in a resting state and inhibit the generation of NO by macrophages in an activated state (polysaccharides and various combinations of polysaccharides with saponins) [41, 42].

Only one study concerned immunocompromised macrophages, derived from macrophages isolated from the dialysate of dialysis patients with renal failure. The results showed increased ability of macrophages to generate NO in the presence of LPS induction after Huangqi injection was administered to patients with renal failure for 9 days [50].

## 4. Discussion

In China, the estimated prevalence of DM in adults aged 18 and older is 11.6%, equating to about 114 million patients [51]. A study indicates that diabetes-related CKD has become the main substantial effect on the observed spectrum of CKD [52]. Clinicians are facing the challenge of how to effectively control the occurrence and progression of DN.

The recommended therapeutic regimens for DN worldwide include controlling blood glucose and hypertension and reducing urinary albumin and blood lipids. In recent years, attempts to treat DN with TCM in combination with western medicine in China have achieved some efficacy. In a national basic research and development project, the efficacy and safety of conventional western medicine combined with TCM in the treatment of DN were evaluated by a multicenter, prospective cohort study, to investigate syndrome-based use of TCM for DN. The results showed that the rule of prescription of TCM was based on supplementing *qi* and nourishing *yin* and promoting blood circulation to remove blood stasis, for which Huangqi was used most frequently [53]. Based on this research, the results retrieved from CNKI showed that simple recipes, compound recipes, and other different formulations of Huangqi all have therapeutic effects on different stages of DN, and the majority of the papers concern Huangqi injections.

Huangqi injection was initially used clinically to treat hepatitis B in 1979 [54], and now is widely used in the treatment of leukopenia [55], viral myocarditis [56], and DN [7–28]. The quality standard for preparation of Huangqi injection, revised by the China Food and Drug Administration in 2002, specified that the amount of Astragaloside IV should be above 0.08 mg/mL [57]. Since Huangqi polysaccharides have many reported pharmacological effects, some of the existing studies investigate the molecular weights and distribution of polysaccharides in Huangqi injection, to provide further basis for the improvement of the quality standard for Huangqi injection [58]. A quantitative assay for simultaneously measuring saponins, such as Astragaloside IV, and flavonoids, such as calycosin and formononetin, contained in Huangqi injection is also being further improved [59, 60]. A pharmacokinetic study on Huangqi injection combined with other drugs such as gliquidone has also provided further reference and basis for the clinical use of Huangqi injection [61]. An effective usage and management system has been established for Huangqi injection with respect to clinical dosage, course of treatment, monitoring of adverse reactions, and so forth through more than 30 years of formulation standardization and gradual improvement of the measurement of various effective ingredients.

The use of Huangqi injection for the treatment of DN was first reported in 1998 [62]. According to our research, Huangqi injection is mainly used on stages III and IV. After many years of multiple RCT observations, meta-analyses as a secondary evaluation can help us more accurately and objectively assess the therapeutic effects of Huangqi injection. Five meta-analyses related to stages III and III-IV were chosen in this paper (Figure 1).

Microalbuminuria occurs in stage III patients, and clinically stage III is normally called the “early stage” of DN. Persistent proteinuria, 30–300 mg/24 h, is a main feature in this stage [1]. If an effective intervention is delivered in this stage, the progression of DN is likely to be delayed or even reversed. This could explain why Huangqi injections are used far more in stage III than other stages. Research showed that Astragaloside IV improved proteinuria, UARE, and BUN in the rat STZ-induced model of diabetes [3]. In Table 2, in total, 70 RCTs and 4 CCTs in stages III and III-IV also showed improvement in indicators including 24-hour urinary protein, UARE, and BUN, and the therapeutic effect of Huangqi injection plus a conventional therapy was better than that of the control group receiving the conventional therapy alone [8–12].

The pathogenesis of DN involves many aspects, such as oxidative stress and immune inflammation. It is considered that macrophages play an important role in the development and progression of DN [5, 6]. In a mouse model of renal ischemia-reperfusion injury (IRI), macrophages were found to highly express iNOS in early IRI, which induced the apoptosis of renal tubular epithelial cells [63].

LPS is a potent inducer and activator of iNOS of macrophages to generate NO. The effect of Huangqi on the generation of NO by macrophages upon activation has been a focus of studies. The 14 articles [29–42] incorporated into this study separately reported that Huangqi extracts and the active ingredients thereof, including polysaccharides, saponins, and total flavonoids, had inhibitory effects on the generation of NO by normal macrophages induced by LPS. Three of these studies simultaneously reported that Huangqi can inhibit the LPS-induced generation of TNF-*α* [29, 30, 38], another specific indicator for activation of macrophage iNOS. The studies above showed that Huangqi inhibited the generation of NO by inhibiting LPS-induced iNOS activity.

Some animal experiments supported the view that the therapeutic effect of Huangqi injection on early DN might be achieved by inhibiting the activity of iNOS. Renal blood flow and glomerular filtration rate were significantly increased in diabetic mice after 4 weeks of STZ induction, with increased expression of iNOS in the renal cortex and medulla. The expression of iNOS was significantly decreased after administration of Huangqi injection [64].

A clinical study showed that after administration of Huangqi injection to patients with renal failure, the secretion of both NO and TNF-*α* from the macrophages isolated from their dialysate was enhanced upon LPS induction [50]. This study suggests that after Huangqi is administered to DN patients with end-stage renal failure, their immunocompromised macrophages in an LPS-activated state may present different functions from normal macrophages in an activated state.

DN is a progressive, chronic metabolic disease. There is still a question as to whether iNOS has a pathogenic effect or protective effect upon activation according to different periods of the disease and different states of the body. A study on different courses of DN in an animal model of STZ-induced DN indicated that iNOS might have different effects in different stages of the disease: in a model of very early stage DN induced by STZ for one week, glomerular hypertrophy and high filtration were associated with the high expression of insulin-like growth factor-1 (IGF-1) in the kidney. It was confirmed that the increase in IGF-1 was associated with an increase in NO, the source of which might be eNOS and iNOS [65].

A number of studies also demonstrated the protective effect of iNOS during the progression of DN. A study in a model of chronic DN for 40 weeks showed that iNOS-derived NO modulates glomerulosclerosis and tubulointerstitial fibrosis in chronic STZ nephropathy [66]. The drugs for treating DN such as pentoxifylline could exert therapeutic effects by increasing the expression of iNOS protein in the kidneys of a mouse model of STZ-induced DN [67].

Of the studies on Huangqi regulation of iNOS activity shown in Table 3, two studies compare the effects of Huangqi polysaccharides and polysaccharides plus saponins on normal macrophages in a resting state and macrophages activated by LPS induction. The results showed that Huangqi polysaccharides and saponins could not only inhibit the production of NO in LPS-induced macrophages but also induce macrophages to generate NO [41, 42]. Seven papers incorporated into this article reported that Huangqi could induce macrophages to generate NO and were focused on polysaccharides and saponins [43–49].

The results of a mechanistic study showed that Huangqi polysaccharides could significantly induce RAW264.7 cells to release NO and enhance iNOS activity. The nuclear factor-kappa B (NF-*к*B) cell signaling pathway was involved in the induction of NO generation and TNF-*α* secretion in macrophages by Huangqi polysaccharides, but NF-*к*B inhibitors did not completely inhibit the induction by Huangqi polysaccharides, suggesting that the NF-*к*B cell signaling pathway may not be the only pathway for this function [45]. Huangqi polysaccharides could induce the generation of NO and improve the phagocytosis of macrophages, which could be blocked by iNOS inhibitors [46]. The studies above suggest that Huangqi can increase the generation of NO by inducing the expression of iNOS in macrophages, thereby exerting pharmacological effects.

It is known that NO exerts biphasic and often antagonistic effects in many processes, depending on factors such as the local tissue concentrations and cell types and the intensity and duration of the inflammatory phase where iNOS is initially produced [68]. Therefore, our first hypothesis is that the pathogenic or protective effect of iNOS generated upon induction may be related to different pathological stages of the same disease and different states of the same patient. The role of iNOS in the initiation, progression, and renal failure of DN still needs to be further clarified.

At present, we are interested in different effects of Huangqi on macrophages in different states: activated or resting and normal or compromised, as shown in the existing literature. Huangqi has different effects on the generation of NO by normal and compromised macrophages upon iNOS activation; it also has different effects on iNOS of macrophages in a resting state and an activated state. Based on the biphasic regulating effects of Huangqi on the generation of NO by macrophages *in vitro*, a second hypothesis is proposed: the therapeutic effects of Huangqi on different stages of DN may be due to effective intervention with some processes of the disease, such as activation or inactivation of iNOS in macrophages and increase or decrease in NO production, at different stages of DN, and at different states of the patient, thereby reversing the imbalances of the disease condition.

The existing preliminary studies support the clinically therapeutic effects of Huangqi on different stages of DN. On the basis of the accumulation of clinical data, clinicians in China are exploring how to obtain high-quality RCT research reports according to the CONSORT standard including standardizing randomized studies, establishing endpoint measures, and particularly strengthening the flowchart of subjects [69].

The pharmacological studies on treatment of DN with Huangqi by regulating the iNOS activity of macrophages are mostly based on *in vitro* experiments, and more *in vivo* studies are required. A recent *in vivo* study suggested that Astragalus polysaccharides may modulate the immunity of the host organism through activation of toll-like receptor- (TLR-) 4 mediated myeloid differentiation factor 88-dependent signaling pathway [70]. Another report suggests that Astragaloside IV might have anti-inflammatory effects *in vivo* by inhibiting the TLR4 signaling pathway [71]. The research showed that blocking TLR4 suppressed LPS-induced iNOS expression, and its role in kidney disease is being explored [72]. Based on this related research, further *in vivo* studies on iNOS/TLR4 pathway involvement might assist our understanding of the role of macrophages in the clinical mechanism of the effect of Huangqi on DN.

Following renal injury and repair in the different stages of DN, macrophages have been shown to exhibit critical regulatory activity. Disturbances in macrophage function can lead to aberrant repair, with uncontrolled inflammatory mediator and growth factor production [73]. Along with the development of phenotypic and functional changes in macrophages, clinical observations, and pharmacological research into Huangqi, further evidence is required to understand the possible biphasic or antagonistic effect of Huangqi on the regulation of macrophage differentiation and polarization, to clarify the roles of Huangqi and macrophages and their interaction in kidney disease.

## Figures and Tables

**Table 1:** Mogensen's criteria for stages of DN [1].

Stage	Features
I	Hyperfunction, hypertrophy.
	Increased urinary albumin excretion (microalbuminuria).
II	Morphologic lesions without clinical signs of disease. Increased GFR.
	Poor diabetic control or exercise increase microalbuminuria.
III	Incipient DN. Persistent proteinuria (30–300) mg/24 h. Microalbuminuria slowly increasing over the years. Increased GFR.
IV	Overt DN. Persistent proteinuria (>0.5 g/24 h). Untreated hypertension leads to decreased GFR.
V	End-stage renal failure with uremia due to DN.

*Note*. DN: diabetic nephropathy; GFR: glomerular filtration rate.

**Table 2:** Analyses of clinical studies on treatment of different stages of diabetic nephropathy with Huangqi *(Astragalus membranaceus)* injections.

Stage	Number of cases		Study design	Main improvement indicators	Publication year
	Control group	Treatment group			
I-II [7]	13	15	RCT	UAER, TGF-*β*1, HbA1c, C-IV	2004
III [8–11]	1644	1724	4 metas, including 49 RCTs	UAER [8–11], 24-hour urinary protein [8–10], Scr [8, 10, 11], BUN [8, 10], TG [8, 9], TC [8, 9], FBG [9, 10]	2013 (2004–2012)^∗^
					2013 (2005–2011)^∗^
					2014 (1998–2012)^∗^
					2017 (1998–2015)^∗^
III-IV [12]	859	945	Meta, including 21 RCTs, 4 CCTs	24-hour urinary protein, Scr, BUN, CCr	2011 (1999–2006)^∗^
IV [13–17]	199	256	5 RCTs	24-hour urinary protein [13–17], Scr [14, 16], BUN [14, 15], CCr [13, 17], TG [13, 16, 17], TC [13, 16, 17]	2000–2015
V [18–28]	120	152	11 RCTs	Scr [18, 20, 22, 23, 25–28], BUN [18, 22, 23, 25–28], CCr [22, 26, 27]	1997–2016

*Note*. ^∗^Publication year of cited paper of RCT or CCT. UAER: urine albumin excretion rate; TGF-*β*1: transforming growth factor *β*1; HbA1c: glycosylated hemoglobin; C-IV: type IV collagen; Scr: serum creatinine; BUN: blood urea nitrogen; TG: glycerin trilaurate; TC: total cholesterol; FBG: fasting blood glucose; CCr: creatinine clearance rate.

**Table 3:** Number of papers on Huangqi (*Astragalus membranaceus*) regulation of inducible nitric oxide synthase activity in different states of MΦ.

Sample type	On resting MΦ	On LPS-activated MΦ	On resting and LPS-activated MΦ	On LPS-activated immune compromised MΦ
Polysaccharides	6 [43, 45–49]	2 [29–30]	1 [41]	
Saponins	1 [44]	1 [33]		
Total flavonoids		1 [32]		
Extracts		8 [31, 34–40]		
Polysaccharides and saponins			1 [42]	
Injection				1 [50]

*Note*. On resting MΦ: effects of Huangqi on resting MΦ; On LPS-activated MΦ: effects of Huangqi on MΦ after LPS activation; On resting and LPS-activated MΦ: effects of Huangqi on resting MΦ and LPS-activated MΦ; On LPS-activated immune compromised MΦ: effects of Huangqi on immune compromised MΦ after LPS activation; MΦ: macrophages; LPS: lipopolysaccharide.
